# Monthly Summary

**Published:** 1895-11

**Authors:** 


### Monthly Summary.

### PEROXIDE OF HYDROGEN.

BY J. PARKER, PH. G., M. D., ST. LOUIS, MO.

Published by the Annals of Opthalmology and Otology, of St. Louis, Mo., April 1895.

(Abstract from *The Times and Register*, June 8th, 1895.)

I have used peroxide of hydrogen quite extensively for cleansing discharging ears, the nasal and accessory cavities, and have tried all of the brands of the preparation in the market, and once thought one manufacturers make as good as that of another, bought the cheapest as a matter of economy, but recent experience has taught me that the difference in quality is greater than the difference in price. After an unpleasant experience with a solution of peroxide of hydrogen which severely injured the mucous membrane, I bought and examined, chemically, a bottle of each preparation of $H_2 O_2$ in the market, and was surprised to find so much difference. Some are useless, and others worse than useless because they contain too little oxygen and too much free acids (phosphoric, sulphuric, hydrochloric). I now order Marchand's (medicinal) exclusively because I find it contains the desired quantity of available oxygen and not enough free acid, to be objectionable, and its keeping properties are all that could be desired.

By inquiry I learn that Marchand's is the preparation that is used by almost all surgeons, and it is considered by them the standard.

—My personal experience with peroxide of hydrogen confirms entirely the statement of Dr. J. P. Parker, I have used exclusively Marchand's brand until lately, when I experimented with hydrozone. Then I gave up entirely the use of peroxide of hydrogen and use hydrozone on account of its strength, which cannot be compared with any other brand, even Marchand's. I must say that the results which I obtained with hydrozone are most gratifying.—ED.

---

WEDGING TEETH WITH RUBBER.—This is still probably the most common way of obtaining the necessary space for making proximal fillings, and is the means of inflicting much unnecessary pain. The practice of inserting the rubber to be worn for days, and sometimes weeks, before operating on the teeth, is cruel, barbarous and unscientific. The object is just as thoroughly accomplished in a few hours, when gutta-percha may be pressed tightly between the teeth that have been forced apart, which will retain the separation and allow all peridental disturbance to heal.

Our practice for years has been to have the patient adjust the rubber early in the morning, and report to us at five o'clock in the afternoon for the gutta percha substitute. The thickness of the rubber supplied the patients is determined by the space required. The gutta-percha is worn for ten days or two weeks, when all soreness has entirely disappeared. The gutta-percha serves the double purpose of retaining the space and pressing away the gum from a cavity which extends well down toward the cervical margins. The cavities are partially prepared, the dam applied, and a separator adjusted to hold the teeth firm during the introduction of the fillings. We know of no more satisfactory means of separating temporarily, and none less free from pain or danger.—*Register.*

---

PULP-PROTECTION.—The writer says that the floor of the cavity in a carious tooth sometimes has a place where the wall of the pulp chamber has but a thin septum between it and the cavity-floor, and the pulp must be protected from thermal shock or irritation. A suitable sized disk of rubber-dam is very good for this purpose. When the cavity is formed and dried for filling, lightly touch the cavity-floor and walls with a very little mastic varnish. Place a small disk of dam over the cavity-floor; mix some cement, suitably soft; put a little on the center of a second disk and place its cement side on the first one. Then spread the cement under the second disk with a ball burnisher to completely cover the cavity floor and partially cover the cavity-walls. After the cement has set, the cavity can be filled with whatever material as preferred. The pulp will be doubly protected by the two rubber disks, and the second can be shaped with scissors to suit the cavity. Thin gutta-percha or paraffin-paper disks, as well as those of vulcanizable rubber may be thus used, and any are preferable to metal disks. When a cavity is filled wholly with cement, a rubber disk, pressed around with a ball burnisher, will carry the cement against the cavity-walls and leave it there; a plugger sometimes carries some cement out with it. An oiled mica matrix, because of its thinness, smoothness, flexibility, resistance to acids, shapibility with scissors, and cheapness, makes an excellent readily-applied and removed cavity-wall. It may be fastened around the tooth with gutta-percha, and if adjusted carefully, no subsequent finish will be required. As the mica matrix is very elastic and frail, care should be taken that it does not spring away from the tooth, and that no broken pieces are left between the teeth.—W. Storer How, D. D. S., in *Cosmos*.

---

TRANSMISSION OF CHOLERA BY THE HOUSE FLY—Craig (*Medical Record*) has made some careful experiments by feeding common house flies with a fresh boillon culture of the cholera sprillium. At the end of three days they seemed uninjured, and when the intestinal contents were removed, with every precaution to prevent contamination from the exterior of their bodies, the nature of which was confirmed by culture experiments and the obtaining of the red culture reaction by the addition of acids to beef-tea culture. If the common house fly is able, without injury to itself, to carry and transmit the cause of cholera, its agency in the extention of epidemics is probably important and may be difficult to combat.—*Philadelphia Polyclinic.*

VARNISHING CAVITIES.—The writer says that owing to the incompatibility of tooth-substance and the materials used for filling teeth, it is best to interpose some substance between the material and the dentine, and he recommends a clear resin such as damson, dissolved in chloroform. It acts as a non-conductor of thermal changes, as well as an insulator against electrical influences. It is not readily soluble in the fluids of the mouth. Being transparent, no discoloration is shown when used where enamel walls are thin; in fact, it prevents discoloration of the tooth from oxidation when an oxidizable amalgam is used. It is helpful in starting large gold fillings, holding the first cylinders firmly to the dentine, and lessening the danger of the fillings coming out. While it does not by any means supply all the anchorage needed, the varnish does away with the deep retaining pits.—W. G. Browne, in *Items of Interest.*

---

Cleaning the teeth should be the first work of the dentist instead of the last. It will not only better reveal defects and enable us to do better work, but prevent the necessity of working in filth. Every particle of tartar should be removed and every tooth scrupulously polished. And such services should be paid for quite as liberally as any other work.—*Items of Interest.*

---

A BROKEN PLATE-HOLDER.—A good method for holding a broken rubber plate in position while it is being waxed up, is to take a round tin box four inches in diameter and two inches deep, with a perforated bottom. Fill this box nearly full of very fine shot. Place the pieces of broken plate in position as they should be, then press down into the shot, drop on the hot wax, and hold box under stream of water to cool. The water will run out through the perforations in the bottom. —*F. E. Buck, in Items of Interest.*

TREATMENT OF CHLOROFORM ASPHYXIA.—At a recent seance of the Paris Academy of Medicine, reported in the "Bulletin of the Academy" Labbe said that in apparent death from chloroform, he was firmly convinced that artificial respiration should be presevered in for a long time, and instanced many cases in support of this advice, particularly one in which he had kept up artificial respiration for twenty seven minutes and was finally rewarded with a remarkable success. Lately, however, he had used rythmical traction of the tongue, suggested by M. Laborde, in the case of a child who soon after the chloroform exhalation, became asphyxiated. Death seemed absolute, the pupils were dilated to the utmost. As soon, however, as he began the rythmatical traction of the tongue, the patient returned to life with marvelous rapidity; it was a genuine resurrection. He believes this method is preferable to artificial respiration, as it is easier and much quicker. Professor Verneuil said that for some time he had used only lingual traction alternating with flagellation of the epigastrum with a wet towel.—*Med. Review.*

---

COBALT AS A PULP DESTROYER.—Dr. C. N. Johnson, of Chicago, in a paper read before the New Jersey State Dental Society, and printed in the December *Cosmos*, advocates the use of Cobalt as a devitalizer of tooth pulps. It is his custom to allow the agent to remain sealed in the cavity one week. In the case of anterior teeth, he usually removes it in twenty-four hours for fear of discoloration, although he believes cobalt will be less likely to discolor a tooth than arsenious acid. The pulp should be well exposed to the action of the agent, and care should be taken in sealing the cavity, preferably with a cement. Dr. Johnson believes in absolute alcohol for dehydrating pulp canals, and considers dryness a *sine qua non* to successful treatment. He is opposed to overtreatment, believing that continued operative interference often results in irritation, which prolongs the disease. He believes in economy of time, both to patient and operator, consistent always with the most thorough work.—*Dominion Dental Journal.*

A CURIOUS case has just been recorded in which an electric current was found to be generated by a plate of artificial teeth. A patient consulted his doctor on account of a severe pain in his tongue. But the sufferer was assured that there was nothing the matter. He then paid a visit to his dentist, who informed him that his teeth were perfectly sound. Being, however, dissatisfied, he called on an electrician whom he knew, and asked him if it were possible that he could have any electricity in his mouth. On examining the teeth, his friend found that two metals were used to fit them to a composition plate. To these metals wire were then attached and connected to a galvanometer. Then the teeth were replaced in the patient's mouth, and the metals moistened with saliva. No sooner was this done than the galvanometer showed quite a large current from so small a source—enough, it is stated, to cause ulceration and severe pain when long continued on so sensitive an organ as the tongue. The plate was covered with an insulating varnish, and thence-forward all the trouble ceased.—*Literary Digest.*

---

INFECTED ROOT CANALS.—Dr. De Marion, of Paris, France, has treated a large number of infected root-canals by filling them with cotton saturated with a 33 per cent. solution of formal (essentially the same as formalin), hermetically sealed in, and in no instance as yet has there been any subsequent inflammatory action.—*Items of Interest.*

---

CORK WEDGES FOR SEPARATING TEETH.—Dr. Denham, of Santiago, states that the application of work for separating is less painful than any other material. It produces neither irritation nor inflammation, and patients can attend to the separating preparatory to a filling themselves. He cuts strips about three-eighths of an inch wide, and with a sharp pen-knife bevels one side to a thin edge, and from these he cuts pieces as required.

DR. GARRETT, of Newkirk, says of pyorrhœa alveolaris that it is disgraceful to hold that by removal of the causes a majority may not be cured. The very first thing to do is to thoroughly cleanse all the teeth at not less than two sittings, finding all the ulcerated points and making records of the same. Any doubtful or carious portion of the process should be removed, and cleansed with peroxide of hydrogen or three per cent. pyrozone, a weak solution of an essential oil such as cinnamon or cassia, to which may be added a few drops tincture iodine. In all these cases the patient has a great deal to do in the treatment and a wash such as listerine should be prescribed for use with the brush. In cases where no calculus exists around the necks, a solution decidedly alkaline, such as bicarbonate or biborale of soda, should be used as a wash. Weak teeth should be supported and in case of lameness cusps ground off to prevent jarring out of place.—*Dominion Dental Journal.*

---

IN resetting or remodeling a rubber plate, Dr. Davenport takes an impression of the mouth, saws away gums and centre of plate, leaving only sufficient rubber to hold teeth in line. This is placed on model and waxed up, tried in and finished in the usual way. In this way considerable time is saved.—*Dominion Dental Journal.*

---

DR. H. A. WALLACE, in a clinic before the Stomatological Club of California, demonstrated his plan of using Saddleback's bicuspids and molars in bridgework. The use of these teeth does away with much that is unsightly in the way of gold cusps in these teeth, besides making a stronger piece of work.

---

NEW ANÆSTHETIC MIXTURE.—Chloroform ten parts ether fifteen parts and menthol one part, used as a spray, is recommended as an excellent and prompt means for obtaining local anæsthesia, lasting about five minutes.—*Medical age.*

ELECTROLYSIS IN ARRESTING EROSION.—It has occurred to me that the diseased glands (labial) can be removed or destroyed by electrolysis. The lips could be easily everted, and by employing a bent electrode, the orifice of the glands could be penetrated, and their decomposition brought about, with a total cessation in the d ischarge of any secretion.—Dr N. P. Brubaker, in *Pacific Druggist and Physician.*

---

TO DEODORIZE IODOFORM.—

R.—Carbolic acid - - 1 part.
Oil of peppermint - - 2 parts.
Iodoform - - - 187 parts.

—"N. Y. Med. Journal.

---

SMOOTH MODELS.—After getting the impression in plaster, give it a coat of shellac, not too thin. After the varnish is perfectly dry, instead of oiling sprinkle powdered soapstone (or French chalk, so called) over the impression; then with a soft brush rub every part of it thoroughly, finally shaking out the surplus. Mix the plaster thin and pour, tapping the cup gently until the plaster commences to set.—*Ohio Journal.*

---

BLEACHING TEETH.—Saturate the dentine with strong sodium peroxide, followed by treatment with dilute hydrochloric acid to neutralize the alkali. Wash with hot water.—E. C. Kirk, in *Dominion Dental Journal.*

---

SPIRITS OF CAMPHOR.—A drop or two placed on the tongue will prevent the nausea in taking impressions.

